# Extracts of Letters on the Vaccine Inoculation

**Published:** 1799-11

**Authors:** 


### *Extracts of Letters on the Vaccine Inoculation, communicated by* Dr. WOODVILLE.

FROM the various accounts in circulation, refpecting the variolæ vaccinæ, I felt anxious for an opportunity of obferving the effects of a difeafe, which the introduction of as a fubftitute for the fmall-pox, feemed to promife fo much benefit to mankind. In the beginning of May laft, I was obligingly fupplied with a piece of thread that had been impregnated with cow-pox virus, by a refpectable furgeon* in the neighbourhood of Birmingham; with this I communicated the difeafe to two patients, whofe cafes are here related. Some time after I had commenced inoculation, I was favored with an additional fupply of vaccine matter by the kind affiftance of Dr. JENNER; with a view of giving it a fair trial, I was particularly careful in the choice of a lancet that had never been ufed for any other purpofe.

On the 11th of May, Thomas and Mary Leicefter were inoculated; the former aged two years and a half, and the latter four months. The thread was inferted into the left arm of each, by means of a fuperficial fcratch, and fecured upon the part with adhefive plaifter.

14th. The arms of both the children appeared inflamed.

15th. The inflammation increafed, and affected the boy's arm more extenfively than the girl's.

16th. The cuticle in each began to rife into a veficle; the boy's arm more forward than the girl's.

17th. The veficles more elevated; the boy was feized at night with fever, and complained of forenefs in the axillæ.

18th. The inflammation of the boy's arm confiderably diminifhed, but the veficle more prominent, and full of a limpid fluid, with about a dozen puftules furrounding the part. At night his fever entirely fubfided.—This evening the girl became feverifh and reftlefs.

19th. A fmall quantity of fluid appeared in the veficle on the girl's arm, with a flight efflorefcence about the tumour, but much lefs than what appeared on the boy's.—The girl had many diftinct puftules on different parts of her body.

* Mr. ADDINGTON, of Weft Bromwich.

20th. She continued indifpofed till night, when all febrile fymptoms difappeared. An elevated fmooth brown fcab remained for a confiderable time upon each of the children's arms, after all difcharge from the part had ceafed. From the arms of the above patients I was fupplied with matter enough to enable me to extend the practice; the progrefs of the difeafe, in general, was fo fimilar, that a minute detail of each cafe would be tedious and unintereiting. Sixty-eight patients, from three months to 22 years of age, paffed fafely through the cow-pox under my care, without *one* alarming circumftance. Thirty-nine had an *eruption*, but only two whofe puftules arrived to a ftate of maturation, and thofe imperfectly. After they were all recovered, I inoculated twelve of them with active variolous matter *without* effect. Their arms appeared inflamed for three or four days, and then gradually got well. I had no opportunity of inoculating a greater number with variolous virus, owing to the parents of the patients being fo well fatisfied with the firft inoculation, from its fimilarity to the fmall-pox, that they thought a fecond trial totally unneceffary. Several of the patients flept with others who had full crops of fmall-pox puftules, without being in the leaft degree affected. The cow-pox patients had the difeafe in every inftance lefs feverely than thofe inoculated with the fmall-pox, although it was communicated at the fame time, in the fame family. A few of the patients inoculated with each difeafe had inflammations of their arms that appeared likely to be troublefome, which were foon fubdued by rubbing into the part unguent. hydrarg. Whenever I have another opportunity of inoculating with vaccine matter, I fhall be difpofed to try Dr. Jenner's method of arrefting the progrefs of the difeafe, by means of mild cauftic applied to the puftule on the arm as foon as I perceive the fyftem affected.

In confequence of the above experiments, and the confidence I have in the "Reports" of the extenfive experience of my ingenious friend, Dr. Woodville, on the fubject, I am decidedly of opinion, that the cow-pox is a certain protection againft the fmall-pox; that it is confiderably more mild in its effects on the human fyftem by inoculation, and under proper management may be fafely introduced at any feafon of the year.

Twenty of the above patients had *no* apparent indifpofition.

Ketley, near Shiffnal, Shropshire,

*Sept.* 11, 1799.

IN compliance with your requeſt I have to inform you that I began to inoculate on the 11th of May, and continued the practice till the latter end of June. The vaccine virus which I received from Mr. ADDINGTON, was originally ſent to him by Dr. PEARSON, of St. George's Hoſpital. On the 8th of June I uſed that ſent me by Dr. JENNER, and no other during the remainder of my practice. The appearance of an eruption on the two firſt patients ſurpriſed me greatly, as well as thoſe ſubſequently inoculated, till I read your "Reports," when my mind was relieved; and after I was favoured with vaccine virus by Dr. JENNER, I was convinced, from the exact ſimilarity of its effects, that what I had received from Mr. ADDINGTON was genuine. At the ſame time I inoculated 50 patients with variolous matter, that I might have an opportunity of obſerving the different effects of the two diſeaſes. And whenever I had it in my power, I inoculated one part of a family with vaccine and the other part with variolous virus. The vaccine patients were ſooner affected, their indiſpoſition in general leſs ſevere, and the diſeaſe of much ſhorter duration than thoſe inoculated with variolous virus. Thoſe few patients whoſe arms were moſt inflamed were of the firſt that were infected, which I attributed to the cold north eaſt wind that prevailed at that time, as they were diſpoſed to become troubleſome ulcers. An eminent ſurgeon in this county, who was employed in inoculating the children of ſeveral reſpectable families with *variolus* matter, poſtponed his practice for a month, on account of the great number of ſore arms that his patients had. After the weather became milder, none of *my* patients gave me trouble, which ſeems as if the ſtate of the atmoſphere, at the commencement of the buſineſs, had ſome influence in producing that effect. As it may afford you more ſatisfaction to have a particular account of the number of puſtules each patient had, I have written it in the ſame form that you did yours in the Reports of your Vaccine Inoculation. The greateſt number of puſtules was generally around the part where the matter was inſerted; they had the appearance that the ſmall-pox has during the eruptive fever, and all (except what I have before mentioned) went off without arriving to a ſtate of maturation.

With ardent wiſhes for a ſpeedy reſtoration of your health,

I remain, dear Sir,

Your obliged and ſincere friend,

KETLEY, *Sept.* 29th, 1799. J. EVANS.

The

The following perſons were inoculated with variolous matter without effect, after being affected with the vaccine:

| | |
|---|---|
| Thomas Barneſley, | Thomas Onions, |
| Ann Barneſley, | Mary Plimmer, |
| Martha Morgan, | Charles Plimmer, |
| Mary Phillips, | Harriet Williams, |
| Eleanor Radcliffe, | William Pinches, |
| John Radcliffe, | John Silveſter. |

A Liſt of thoſe Perſons who were inoculated with the Vaccine Virus.

| | Years of Age. | Months. | Days indiſpoſed | No. of Puſtules. | | Years of Age. | Months. | Days indiſpoſed | No. of Puſtules. |
|---|---|---|---|---|---|---|---|---|---|
| Thomas Leiceſter | 2 | 6 | 1 | 12 | Elizabeth Lloyd | 3 | 6 | — | 3 |
| Mary Leiceſter | — | 4 | 2 | 40 | Ann Roberts | 5 | 6 | 2 | 40 |
| Jane Stevens | 2 | 6 | 2½ | 6 | Mary Roberts | 2 | 6 | 1 | 6 |
| John Perry | 1 | 6 | 1 | 16 | Hugh Thomas | 2 | — | 1 | — |
| Charles Plimmer | 10 | — | 1 | 30 | Elizabeth Cadman | 7 | — | — | — |
| Thomas Plimmer | 15 | — | 1 | — | Morris Cadman | 4 | — | — | — |
| Mary Plimmer | 12 | — | 2 | — | James Cadman | 3 | — | 3 | — |
| Matthew Williams | — | 7 | — | — | Sarah Jones | 4 | — | 3 | — |
| Martha Morgan | — | 9 | 2 | — | William Jones | 2 | — | — | — |
| Mary Roſon, ſen. | 22 | — | 3 | 15 | Thomas Cadman | 1 | — | — | — |
| Thomas Socket | 9 | — | — | — | Ann Jones | — | 6 | 3 | 2 |
| Willam Socket | 5 | 6 | — | — | Ann Barneſley | 6 | — | 2 | 1 |
| Joſeph Socket | 1 | — | 1 | 3 | Thomas Barneſley | 3 | — | 2 | 3 |
| Thomas Onions | 1 | 3 | 2 | 20 | Thomas Pearce | 2 | — | 3 | 1 |
| Benjamin Burrowes | 2 | — | 3 | 50 | Maria Briſcoe | 1 | 6 | 2 | 20 |
| Thomas Cranage | — | 5 | 3 | 18 | Mark Dod | 3 | — | 2 | 1 |
| Jane Jervis | 1 | — | ½ | 100 | Martha Dod | 1 | 6 | 1 | 50 |
| Mary Phillips | 2 | — | 2 | 50 | Thomas Cooke | 3 | — | — | — |
| Harriet Williams | 1 | 3 | — | 10 | Elizabeth Cooke* | 1 | — | 2 | 100 |
| Hannah Aaron | 3 | — | 3 | 2 | Elizabeth Hill | — | 6 | 1 | — |
| John Cooke | 2 | — | 1 | 12 | Ann Trickett | — | 7 | 1 | — |
| Mary Bird | 4 | — | 2 | 20 | Elizabeth Hazle | 3 | — | — | — |
| Elizabeth Aſton | 1 | — | — | 4 | Rebecca Hazle | — | 9 | — | — |
| Mary Roſon, jun. | — | 4 | 1 | 6 | William Onions | 2 | — | — | — |
| John Radcliffe | 4 | — | — | — | Jacob Brooks | 1 | 6 | — | — |
| Eleanor Radcliffe | 2 | 6 | — | — | Maria Brice | 2 | 6 | 1 | 20 |
| Stephen Smith | 2 | 6 | — | — | William Brice | 1 | — | ½ | 30 |
| James Burns | 1 | 6 | — | 8 | John Churm | 4 | — | 1 | — |
| William Dean | 3 | — | 1 | — | Thomas Churm | 2 | — | 2 | — |
| Ann Wright | 1 | 6 | 2 | 5 | James Churm | — | 3 | 1 | — |
| Ann Onions | — | 8 | ½ | 8 | Mary Ann Parker | — | 10 | ½ | 12 |
| Francis Gregory | 7 | — | 2 | 6 | John Hazle | 3 | — | 2 | 100 |
| Elizabeth Gregory | 5 | — | — | — | William Pinches | 1 | 3 | ½ | 3 |
| Robert Ellis | 2 | — | — | 1 | John Silveſter | 14 | — | 2 | — |

* Cutting teeth.

